# Lithium management around delivery: a retrospective observational cohort study

**DOI:** 10.1192/j.eurpsy.2024.673

**Published:** 2024-08-27

**Authors:** M. L. Imaz Gurruchaga, M. Torra Santamaria, K. Langohr, R. Martin-Santos

**Affiliations:** ^1^ Unit of Perinatal Mental Health Clinic-BCN; ^2^Pharmacology and Toxicology Laboratory, Hospital Clinic; ^3^Department of Statistics and Operational Research, Universitat Politècnica de Catalunya; ^4^Psychiatry and Psychology, Hospital Clinic, Barcelona, Spain

## Abstract

**Introduction:**

During the perinatal period lithium is proven effective as maintenance therapy and to prevent postpartum psychosis. Pregnancy affects all aspects of kidney physiology altering the pharmacokinetics of lithium. To minimize the risk of both maternal and neonatal complications around delivery, several authors have provided clinical advice on lithium dosing around delivery: decreasing dose by 30-50%, suspend lithium therapy 24-48 hours before scheduled cesarean section or induced delivery or even discontinuing lithium after first signs of labour.

**Objectives:**

To evaluate the validity of these recommendations by investigating 1) maternal lithium serum concentrations changes around delivery, 2) the lithium trasplacental passage at delivery and 3) the association between neonatal lithium serum concentration at delivery and neonatal outcomes.

**Methods:**

Psychopathologically stable women with a singleton pregnancy (n=66) who used lithium around delivery, were included in this retrospective observational cohort study (HCB/2020/1305). All women were advised to suspend lithium administration at the onset of labour in the event spontaneous deliveries. Study date: demographic, psychiatric, obstetric and neonatal outcomes for each mother-infant pair obtained from the hospital medical records.Lithium serum concentrations were determined by means of an AVL 9180 electrolyte analyzer based on the ion- selective electrode (ISE) measurement principle. Limit of quantification (LoQ) was 0.20 mEq/L.

**Results:**

The most common psychiatric diagnosis was a bipolar disorder type I (n=54, 90%). Forty mothers (61%) were on lithium monotherapy. Mean (SD) umbilical cord and intrapartum maternal lithium serum concentration was 0.59 (0.13) mEq/L and 0.55 (0.13) mEq/L respectively. There was a strong positive correlation between umbilical cord and maternal lithium serum concentrations (Pearson correlation coefficient 0.95 (95%IC: 0.91,0.97). In a subsample (N=22) a paired t test indicates that the maternal serum lithium concentrations at delivery were significantly lower (mean difference=0.19 mEq/L, 95%CI=0.13-0.25) than those during obtained the day before delivery hospitalization, after a mean (SD) of 31.29 (±11.92) hours (SD=11.92) have elapsed since the taking the last dose of lithium prior to delivery. Four women (6%) relapsed early postpartum.There were no significant differences between lithium monotherapy (N=18/40) and polytherapy (N=11/26) groups with regard to acute neonatal complications (p>0.05). The only acute neonatal complication associated to umbilical cord lithium serum concentration was hypotonia [0.712 (0.298) vs. 0.534 (0.214) (F=5.065; df=1,60; p=0.028)].

**Conclusions:**

When lithium is used around delivery, maternal and neonatal well-being can be maximized by maintaining maternal serum lithium concentrations at the minimal effective level and discontinuing briefly when presenting to hospital for delivery.

**Disclosure of Interest:**

None Declared

